# An Energy-Efficient Evolutionary Clustering Technique for Disaster Management in IoT Networks

**DOI:** 10.3390/s20092647

**Published:** 2020-05-06

**Authors:** Morteza Biabani, Hossein Fotouhi, Nasser Yazdani

**Affiliations:** 1 School of Electrical and Computer Engineering, College of Engineering, University of Tehran, Tehran 14395-515, Iran; yazdani@ut.ac.ir; 2 School of Innovation, Design and Engineering, Mälardalen University, 721 23 Västerås, Sweden; hossein.fotouhi@mdh.se

**Keywords:** IoT networks, energy-efficient clustering and routing, disaster management, non-uniform distribution of events, evolutionary algorithms, tree encoding, forest fire

## Abstract

Wireless Sensor Networks (WSNs) are key elements of Internet of Things (IoT) networks which provide sensing and wireless connectivity. Disaster management in smart cities is classified as a safety-critical application. Thus, it is important to ensure system availability by increasing the lifetime of WSNs. Clustering is one of the routing techniques that benefits energy efficiency in WSNs. This paper provides an evolutionary clustering and routing method which is capable of managing the energy consumption of nodes while considering the characteristics of a disaster area. The proposed method consists of two phases. First, we present a model with improved hybrid Particle Swarm Optimization (PSO) and Harmony Search Algorithm (HSA) for cluster head (CH) selection. Second, we design a PSO-based multi-hop routing system with enhanced tree encoding and a modified data packet format. The simulation results for disaster scenarios prove the efficiency of the proposed method in comparison with the state-of-the-art approaches in terms of the overall residual energy, number of live nodes, network coverage, and the packet delivery ratio.

## 1. Introduction

The Internet of Things (IoT) includes a large number of distributed nodes which are capable of sensing and interacting remotely [1,2]. Wireless Sensor Networks (WSNs) are classified as the major players of IoT networks and provide a wireless connection between constrained devices. In IoT-based WSNs, nodes transmit collected data (such as environmental information) to a central destination known as a sink node through a wireless channel [3,4]. Due to the recent advancements in hardware design, sensor devices are tiny and have a low cost, enabling their deployment in harsh environments such as nuclear power plants and forests [2]. However, sensor devices have very limited battery power, requiring the design of energy efficient algorithms [2,5]. Limitations in energy resources have a direct impact on the application performance, as sensors with depleted batteries can cause network disconnections and consequently packet losses [6,7,8,9]. In a multi-hop sensor network, it is crucial to devise algorithms and mechanisms in such a way to select the optimal route for data transfer to reduce energy consumption [2,5,10,11].

There are several IoT applications that are classified based on their requirements and specifications. Safety-critical IoT applications have very strict requirements in terms of reliability and timeliness. This class of applications is defined as a set of behaviors and actions which are taken to prevent, control, and overcome disasters (e.g., fires and earthquakes) [2,6,9,12]. We define disaster management as techniques that deal with devising algorithms and protocols that provide an enhanced quality of service in safety-critical applications. Forest fire detection is an example of a safety-critical application; an unexpected fire can cause a great deal of environmental and ecological damage while threatening human life [13,14]. Regardless of the cause of the crisis, disasters such as earthquakes and fires create an emergency position and have irreparable effects on human society. Recently, due to the escalation of disasters, researchers have focused on the design of warning and remote control systems [15]. IoT protocols and algorithms provide more energy-efficient, portable, and scalable solutions to various disaster problems [2]. This technology provides an open opportunity for the development of disaster-resilient smart environments such as smart community solutions. In this paper, the proposed method uses IoT networks to remotely monitor forest fires. If a disaster occurs, an early warning system is supposed to perform disaster reporting, connecting to a central control unit that takes preventive actions. Due to the weather situation, forest fires may undergo sudden changes that make the monitoring process more complex as the location and spread of fire varies over time [13]. Furthermore, due to the non-uniform distribution of events and the uncontrolled behavior of fire, sensor nodes are unlikely to have a uniform energy consumption. There are some works in the literature focusing on clustering and routing approaches for forest fire management [13,16,17,18,19,20]. Most of these works are focused on rapid fire detection and have neglected the energy efficiency of the network as one of the key elements [17,19]. To the best of our knowledge, existing research works into WSNs in disaster management cover a uniform distribution of events [18,19,21]. These methods cannot be practically efficient in disaster management with a non-uniform distribution of events, which is more common in real experiments. Thus, disaster management using IoT-based WSNs requires an appropriate routing to be created from the cluster heads (CHs) to the sink in the disaster area.

Clustering is one of the techniques which is widely used for hierarchical routing [22,23,24,25,26]. The purpose of clustering is to search for and find optimal CH nodes. Due to the complexity of searching for optimal nodes as CHs, clustering in WSNs is time-consuming. In fact, clustering is known as an NP-hard problem [22,23,27]. However, previous studies show that during cluster head selection with traditional algorithms, it is necessary to employ evolutionary algorithms to increase network efficiency [6,27,28,29,30].

In this paper, the energy-efficient cluster head selection and routing (ECHSR) method based on evolutionary algorithms such as Particle Swarm Optimization (PSO) and the Harmony Search Algorithm (HSA) are proposed for disaster management. Although PSO has advantages such as being highly dynamic, featuring rapid convergence, and escaping local optimal solutions [23,27,29], in the face of high-dimensional optimization, it can be very difficult to search for all the possible solutions in every part of the search space [29,31,32]. Having the advantage of a high-search ability, the HSA is capable of solving this problem. Moreover, PSO is confined to exploration and HSA to exploitation, and it is wise to balance exploration and exploitation. Therefore, a hybrid PSO–HSA method will increase energy efficiency more than using these techniques separately [29]. Some studies indicate that meta-heuristic algorithms such as HSA and PSO cannot lead to maximum performance as it is necessary to overcome premature convergence to provide the optimal balance between exploration and exploitation [31,33,34]. Therefore, the modified hybrid PSO–HSA method can be a suitable choice for clustering in forest fires.

This study is conducted in two main phases: (i) devising an improved hybrid PSO–HSA-based CH selection method, and (ii) developing PSO-based routing with modified tree encoding. In the formulation of the fitness function of the clustering phase, new criteria entitled “energy efficiency”, “network closeness” and “network coverage” are considered. In addition to solving fitness function formulas, we use the adaptive weighted sum (AWS) method, which is based on Pareto optimization [28,35,36,37,38]. Furthermore, for the routing phase, we use the PSO to select relay nodes (RNs) and send packets from CHs to the sink node. However, the PSO may increase the number of invalid paths, which is not suitable for this application [7,27,39]. Thus, in order to reduce the invalid paths for our application, we provide an optimal encoding tree based on a new type of packet format. The simulation results demonstrate that, compared to the state-of-the-art, the proposed method increases the network lifetime by expanding the range of network coverage. The contributions of this paper are summarized as follows:

- Providing an energy-efficient method for clustering and routing in WSNs while considering the characteristics of disaster areas (with a non-uniform distribution of events).
- Proposing an improved hybrid PSO–HSA-based CH selection method with a novel fitness function including energy-efficiency, cluster closeness, and network coverage criteria.
- Utilizing the PSO to select relay nodes based on multi-hop routing with a fitness function including energy-efficiency and communication link quality criteria.
- Using the adaptive weighted sum (AWS) method as a mathematical model to solve the fitness function based on the multi-objective optimization problem (MOP) so that a lower optimization cost can be obtained.
- Developing a new type of packet format which is adapted to use in disaster applications; furthermore, modifying the PSO encoding priority-based routing to build an optimal routing tree with non-uniform events.
- Overcoming the state-of-the-art approaches in terms of their average energy consumption, the number of live nodes, network coverage, and packet delivery ratio with various sink locations in disaster scenarios.

The rest of the paper is organized as follows. A review of the related works is given in Section 2, which is then followed by our proposed method in Section 3. The simulation results and evaluations are described in Section 4. Finally, Section 5 concludes the work and explains the possible future works.

## 2. Related Works

In [22], the authors introduced a low-energy adaptive clustering hierarchy (LEACH), which is a popular distributed heuristic method. Clustering in this method is dynamic, random, probabilistic and periodic. LEACH determines a threshold for CH selection. It produces a random number between [0, 1] to select a CH in that period. One of the disadvantages of the LEACH protocol is the random selection of CHs, because all nodes can be eligible to be a CH. Another hierarchical heuristic method, named the threshold-sensitive energy efficient sensor network protocol (TEEN), was proposed to improve energy consumption using the two-layer structure for clustering [40]. In this method, two thresholds are applied: (i) the hard threshold (HT) and (ii) the soft threshold (ST). Each CH sends its threshold values to its members. Both thresholds are defined in order to reduce the number of transfers over the routing periods and thus to reduce energy consumption in the process. In [40], CH selection is based on probability, and the distribution of CHs will be non-uniform. The TEEN algorithm has improved clustering stability and energy efficiency compared to LEACH. Its main drawback is that nodes will never establish communication if the thresholds are not reached.

To overcome the limitations of heuristic algorithms, such as inefficiently exploring the search space, meta-heuristic algorithms have been proposed for optimal CH selection [27,29]. Access to every portion of the entire search space over each period is considered to be one of the most important criteria for increasing efficiency in these algorithms, which leads to optimal or near-optimal solutions. Meanwhile, evolutionary algorithms (EAs) such as PSO and HSA are some of the most famous population-based algorithms, and their fitness function determines the quality of the solutions [32].

The authors in [23] proposed an evolutionary PSO for energy-aware CH selection by combining criteria such as the Euclidean distance between the nodes and CHs and the remaining energy from particle energy to node energy, allowing an evaluation of the issues related to the degree of the nodes and the number of CH hops required to reach particle sinks. An analysis of the results proved that the inclusion of energy criteria in the fitness function plays a crucial role in energy efficiency. In [23], CH selection was based on probabilities, and the CH distribution was uniform. This method also focused more on the exploration stage than exploitation. In [41], the authors presented an evolutionary HSA inspired by music for CH selection. Musicians try to select better notes in order that their music can be heard better, which forms the concept of presenting the harmony fitness function. In other words, the best set of CHs is selected. The authors focused on the two criteria of energy (HE) and distance (HD) for harmony evaluation. The fitness function of the Hoang schema is as follows:

(1) $$f_{HS}={\;\;\;\alpha \;\;\;}_{1}\times \mathrm{HD}+{\;\;\;\alpha \;\;\;}_{2}\times \mathrm{HE}$$

HD and HE are calculated according to Equations (2) and (3):

(2) $$\mathrm{HD}=\mathrm{ma}x_{k}\left\{\sum_{\forall n_{i}\in CM_{k}}\;\frac{d\left(n_{i},CH_{k}\right)}{\left\|\mathrm{CM}\right\|}\right\}$$

(3) $$\mathrm{HE}=\frac{\sum_{i=1}^{N}E\left(n_{i}\right)}{\sum_{j=1}^{k}E\left(CH_{j}\right)}$$

It is noteworthy that α is a weight factor, and its value lies between 0 and 1. The furthest Euclidean distance of each node involved in a cluster to its CH ($CH_{k}$) is evaluated according to HD, in which the number of the members in each cluster is presented by ‖CM‖. HE is also evaluated by dividing the sum of current energy in all network nodes $E(n_{i})$ by the sum of the current stage of CH energy $E(CH_{j})$. In [41], CH selection is random and CH distribution is non-uniform. Additionally, HSA is focused more on the exploitation stage than exploration. In [27], the authors introduced a two-tier PSO protocol for clustering and routing in WSNs entitled TPSO-CR. The three measures used in the fitness function of the clustering were network coverage, the remaining energy of nodes, and the link communication quality. Moreover, the two criteria utilized in the fitness function of the routing were energy-efficiency and link communication quality, which are described in the following. In this paper, the energy criteria are used in an attempt to reduce the number of the sender nodes; by doing so, the residual energy will be saved optimally. Furthermore, tree encoding is facilitated by applying PSO to construct optimal multi-hop routing and determine the Relay Nodes (RNs). Thus, Equation (4) is defined in a more energy-saving state (ER1) as follows:

(4) $$ER_{1}=\frac{\left|\left|\mathrm{RNs}\right|\right|}{\left|\left|\mathrm{actCH}\right|\right|}$$

ER1 divides the number of RNs by the number of active CHs in the corresponding period ‖actCH‖. In fact, Equation (4) reduces the number of active nodes. In addition, the RN with the highest level of energy should be offered as the sender node. ER2 adheres to that concept, as per Equation (5):

(5) $$ER_{2}=\frac{\sum_{i=1}^{N}E\left(\mathrm{nod}e_{i}\right)}{\sum_{j=1}^{\mathrm{RN}}E\left(RN_{j}\right)}$$

Similarly, ER2 divides the sum of the energy of all nodes by the sum of the RN energy and moderates the energy consumption. Next, maximizing link quality (LiQ) between RNs in the multi-hop routing tree leads to the maximization of the packet delivery ratio (PDR). Hence, the worst communication link quality must be avoided in all sub-branches of the routing tree. Equation (6) is calculated according to this objective as follows [27]:

(6) $$\mathrm{LiQ}=Max_{rn}\sum_{\forall rn_{x}\in \mathrm{RN}}\;\frac{\mathrm{RSSI}\left(rn_{x},rn_{x+1}\right)}{\mathrm{minRSSI}}$$

The received signal strength indicator (RSSI) is used in this paper to evaluate the link quality between the cluster nodes. In [27], the link quality is the ratio of the RSSI between the CMs and their CHs to minRSSI. In Equation (6), it can be observed that we prevent the selection of the worst link quality (minRSSI) as far as possible between two consecutive sender nodes $\mathrm{RSSI}\left(rn_{x},rn_{x+1}\right)$. Hence, In order to maximize the quality of the clusters in terms of LiQ, we must minimize the worst quality of the clusters. So, the fitness function of routing is formulated as follows:

(7) $$\min F_{\mathrm{routing}}={\;\;\;\beta \;\;\;}_{1}\times ER_{1}+{\;\;\;\beta \;\;\;}_{2}\times ER_{2}+{\;\;\;\beta \;\;\;}_{3}\times \mathrm{LiQ}$$

where ${\;\;\;\beta \;\;\;}_{j}\in \left[0,1\right]$. Furthermore, the authors in [29] produced a hybrid method based on PSO and HS algorithms for energy-efficient CH selection. The proposed meta-heuristic algorithm takes advantage of both algorithms, covering a global optimal in the search space (exploration) and going beyond the local optimal (exploitation). Furthermore, the authors were inspired by Equation (1) [41] as regards the fitness function, with the aim of energy-efficiently minimizing the average distance of the normal nodes to the CHs. In PSO–HSA [29], the particles are allowed to move from one region to another area of the search space based on the PSO algorithm. Since PSO encounters optimization limitations in high-dimensional scales, it is difficult to find any possible solution in the entire search space. Therefore, HSA helps PSO because of its high searching capacity. In [42], the authors used the cuckoo algorithm for clustering and employed an improved HSA for multi-hop routing in WSNs. They suggested that uniform energy consumption in the clustering and routing of large-scale wireless sensor networks is one of the main concerns in this area, because, due to traffic load, nodes near the sink suffer from high energy consumption. This paper also proposes criteria such as the energy, node degree, intra distance of clusters and the coverage ratio for the fitness function.

The performances of different clustering protocols in WSNs are summarized in Table 1, from which the following can be concluded: (1) the Hybrid PSO-HSA [29] is more energy-efficient than the cuckoo [42], PSO [23], and HS [41] algorithms; (2) the cuckoo algorithm, in comparison with PSO and HSA, has poor cluster stability, and all of the algorithms suffer from an imbalance of exploration and exploitation; (3) none of the related works into WSNs consider the non-uniform distribution of events, and this is not suitable for disaster application.

## 3. Proposed Method

In this section, first, the network model is explained; then, by determining the AWS method for fitness functions, two main phases of our method are proposed.

### 3.1. Network Model

**Figure 1:**
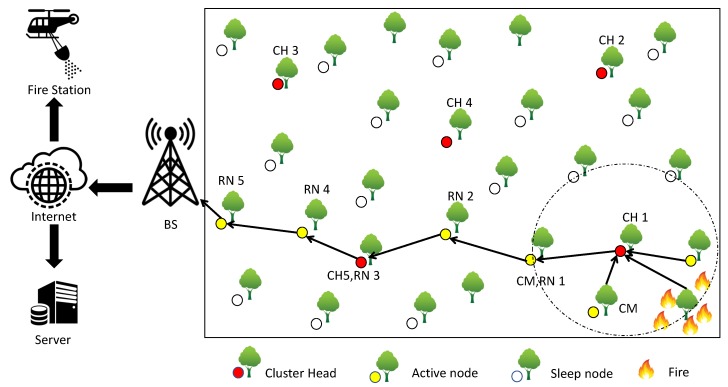
The network topology of a typical WSN network for disaster management.

In our protocol, the nodes are scattered homogeneously and randomly with a uniform distribution in a rectangular field (100 m × 200 m). Figure 1 indicates the overall network model, including a description of the uniform random distribution of the nodes in a disaster application such as a forest fire.

Our method is configured based on the following assumptions, and the location of the sink is considered to be variable according to the evaluation of the algorithm’s efficiency in the target application.

- The nodes are fixed (stationary) in the network.
- Nodes are not location-aware, but the sink is aware of its position.
- All nodes have the same level of energy (E0) initially, and the network is homogeneous [7].
- The minimum RSSI with the value of −97 db is known as the worst received signal [27].
- The number of CHs (k) is considered to be 5% of all nodes (N) [27,29,30].
- Nodes always send their data based on Time Division Multiple Access (TDMA), which is enforceable on IEEE 802.15.4/ZigBee standard. Additionally, we use the Timeout MAC (TMAC) protocol because it is compatible with different sleep schedules in WSNs and provides energy efficiency [27,43].
- The sink is responsible for clustering and routing. Therefore, the proposed method is centralized.
- The energy consumption of the nodes is estimated based on their distance from the sink or CH (by parameter d) [22].

In [22], the authors evaluate the energy consumption model based on the distance between the sender and receiver (d). In this model, the energy dedicated to sending messages is calculated by the length of the messages (L) and the distance between sender and receiver (d) as follows [22]:

(8) $$E_{\mathrm{Tx}}\left(l,d\right)=\left\{\begin{array}{c} L.E_{\mathrm{elec}}+L.{\;\;\;\varepsilon \;\;\;}_{\mathrm{fs}}.d^{2}\;\;\;,if\;\;\;\;\;\;d\leq d_{0}\\ L.E_{\mathrm{elec}}+L.{\;\;\;\varepsilon \;\;\;}_{\mathrm{amp}}.d^{4},if\;\;\;\;\;d>d_{0} \end{array}\right.$$

In (8), the energy consumption to send one bit of data is calculated by $E_{\mathrm{elec}}$. Additionally, sending factors of ${\;\;\;\varepsilon \;\;\;}_{\mathrm{fs}}$ and ${\;\;\;\varepsilon \;\;\;}_{\mathrm{amp}}$ are valued in proportion to the booster model of the sender-radar. In [22], the consumed and dedicated energy of length L (in bits) will be calculated according to Equation (9) to receive each message.

(9) $$E_{\mathrm{Rx}}=L.E_{\mathrm{elec}}$$

It is noteworthy that the initial distance d0 is calculated as follows in Equation (10):

(10) $$d_{0}=\sqrt{\frac{{\;\;\;\varepsilon \;\;\;}_{\mathrm{fs}}}{{\;\;\;\varepsilon \;\;\;}_{\mathrm{mp}}}}$$

### 3.2. AWS Method for LP Formula

One of the most significant challenges in disaster management is decision-making in harsh environments [44]. In these cases, the decision-maker faces different criteria influenced by the internal and external environment. Hence, multi-criteria decision making (MCDM) schemas are considered to be among the best tools to make decisions [36,39]. In MCDM, instead of using an optimality criterion, several metric criteria may be used. Among the methods to solve these problems is the adaptive weighted sum (AWS) method [38]. As the simplest method of MCDM analysis, the AWS method can be used only when all criteria are stated precisely in terms of the same domain. Moreover, it permits a multi-objective optimization problem (MOP) to be converted into a single objective mathematical optimization problem (SOMP) format. The MOP method is formulated as follows [28,37,38]:

(11) $$\mathrm{MOP}:\;\;\;\;\;\;\;\min_{x\;\;\;\;\;\;\;\in \;\;\;\;\;\;\;\;\;\Omega \;\;\;}\;F\left(x\right)=\min_{x\;\;\;\;\;\;\;\in \;\;\;\;\;\;\;\;\;\Omega \;\;\;}\;\left(f_{1}\left(x\right),f_{2}\left(x\right),\;\;\;\;\;\;\;\ldots ,f_{n}\left(x\right)\right)$$

subject to $F\left(x\right):\Omega \to R^{n}$. Multiplying the sum of the fitness functions in the weighted coefficients, the AWS method transforms Equation (11) to the SOMP as per Equation (12) [38]:

(12) $$\min\underset{j=1}{\sum^{N}}\;w_{j}.f_{j}\left(x\right)$$

subject to j∈Ω, where $w_{j}\geq 0,\left(\forall j=1,2,\ldots ,N\right),and\sum_{j=1}^{N}w_{j}=1.$

In this paper, we use the above equation to determine the fitness functions. Equation (12) is also called Pareto optimization. Typically, this kind of optimization gives a unique solution (if $w_{j}\;>0,\;\forall j=1,2,\;\ldots ,\;N$) [38]. As the proposed fitness functions are composed of different criteria and values, it is also essential to normalize the criteria with respect to the Pareto optimization and match them with the weighted coefficients [38].

#### Normalizing the LP Formula

In order to obtain a similar amplitude value of the fitness functions, each criterion of these functions is normalized between [0, 1] [36,37]; all criteria of the fitness function should be in one domain. Normalizing the interval between 0 and 1 requires the Utopia and Nadir points [38]. The Utopia point, which is the lower bound ($S^{U}$) of the function, is modeled as follows:

(13) $$s_{y}^{*}=fo_{y}\left(x^{\left[y\right]}\right)\;\;\;\;\;\;\;\;\;\;\;\;\;\;\;\;\;\;\;\;\;\;\;\;\;\;\;\;\;\;\;\;\;\;\;\;\;\;\;\;\;\;\;\;\;\;\;\;\;\;\;\;\;\;\;\;\;\;\;\;\;\;\;\;\;\;\;\;\;\;\;\;\;$$

where $s_{y}^{*}=\mathrm{argmi}n_{x}\left\{fo_{y}\left(x\right)\;\;\;\;\;\;\;:x\;\;\;\;\;\;\;\in \;\;\;\;\;\;\;\;\;\Omega \;\;\;\right\}$ and subject to $s_{y}^{*}\in R,S^{*}\in R^{Y}$. The Utopia point is $\;\;\;\;\;\;\;S^{U}=\;\;\;\;\;\;\;\;\;\;\;\;\;S^{*}$. The Nadir point, which is the upper bound ($\;\;\;\;\;\;\;S^{N}$) of the function, is modeled as follows [38]:

(14) $$s_{y}^{N}=\max_{1\leq j\leq Y}\;\left(fo_{y}\left(x^{\left[j\right]}\right)\right),\;\;\;\forall y=1,2,\ldots ,Y.$$

If considering the difference in values at points, then $\;{\;\;\;\theta \;\;\;}_{y}=\;\;\;\;\;\;\;\frac{1}{s_{y}^{N}-\;\;\;\;\;\;\;s_{y}^{U}}$. Finally, the normalization equation is calculated as follows [38]:

(15) $$0\leq \frac{fo_{y}\left(x\right)-\;\;\;\;\;\;\;s_{y}^{N}}{s_{y}^{N}-\;\;\;\;\;\;\;s_{y}^{U}}\leq 1$$

According to the above equation, it is possible to calculate values at each round of the simulation for each criterion.

### 3.3. Cluster Head Selection Phase

In the clustering phase, our evolutionary method is described through the contribution of the hybrid PSO–HSA [29] with the difference that the fitness function is modified and improved according to the disaster management conditions. The improved fitness function and stages of clustering will be explained below.

#### 3.3.1. Improved Fitness Function for CH Selection

In order to increase the network lifetime, a set of optimal CHs should be selected; if the number of CHs were too low, nodes would have to increase their communication range to send the information, and so they would consume more energy. However, if the number of CHs were too high, the network would be a single hop, again leading to increased energy consumption. Therefore, it is necessary to determine the optimal criteria of the fitness function based on the application [26]. In this paper, the fitness function in WSN is a multi-objective function that needs to be minimized to reach optimal or near-optimal solutions. Additionally, three criteria have been used to select CHs. First, the energy-efficiency criterion (EE) is included, inspired by Equation (3) of the Hoang method [41], as mentioned above; then, two other criteria are added to the fitness function, which are called cluster closeness (CC) and network coverage (NC).

#### 3.3.2. Cluster Closeness (CC)

To maximize cluster quality, it is necessary to make highly compact clusters to reduce energy consumption [33]. Furthermore, a high density of nodes is a good option for selecting a candidate CH from a region [33] because the energy consumption and internal communication cost of clusters is therefore minimized [14,27,29]. Thus, we attempted to decrease the mean distance of the CH to its neighbors for rapid data transmission. If the density of an arbitrary node x (CH candidate) is the number of single-hop neighbors $\left(nh\left(x\right)\right)$ and the sum of the Euclidean distance of node x from single-hop neighbors $\left(h\left(x\right)\right)$ is $dist\left(x,\;h\left(x\right)\right)$, then the cluster closeness of an arbitrary node x is calculated as follows:

(16) $$\mathrm{Cluster}\;\mathrm{Closeness}\;\;\;\;\;\;\;\left(CC_{x}\right)=\frac{\sum_{y\in h\left(x\right)}\mathrm{dist}\left(x,y\right)\;\;\;\;\;\;\;}{\mathrm{nh}\left(x\right)}$$

A node will be chosen as CH which has a lower value than the other candidate CHs because increasing the $nh\left(x\right)$ value according to [33] is effective in selecting CHs. Similarly, as in Equation (2), the mean Euclidean distance of the CMs with the CHs is minimized.

#### 3.3.3. Network Coverage (NC)

One of the important factors in overcoming the challenges of WSNs is network coverage [15,27]. If the aim is to reach higher scalability, then the network coverage should be increased [27]. Thus, finding a CH for the nodes that have no CHs (non-CHs) can effectively cover the network. In this paper, the network coverage is evaluated as follows:

(17) $$\mathrm{Network}\;\mathrm{Coverage}\;\;\;\;\;\;\;\left(\mathrm{NC}\right)=\frac{\left(N^{*}-K\right)-\sum_{k=1}^{K}\left|\left|CM_{k}\right|\right|}{\sum_{k=1}^{K}\left|\left|CM_{k}\right|\right|}$$

where $\left|\left|CM_{k}\right|\right|$ represents the kth total number of CMs in the cluster, K is the number of CHs, and N* is the total number of nodes (N) minus the number of nodes annihilated in each round. Minimizing the number of non-CHs is perfromed through NC [15,27]; thus, by minimizing the EE, CC, and NC parameters, the best set of CHs is selected. A novel fitness function of the clustering phase is formulated as shown in Equation (18):

(18) $$\min F_{\mathrm{clustering}}={\;\;\;\alpha \;\;\;}_{1}\times \mathrm{EE}+{\;\;\;\alpha \;\;\;}_{2}\times \mathrm{CC}+{\;\;\;\alpha \;\;\;}_{3}\times \mathrm{NC}\;\;\;\;\;\;\;\;\;\;\;\;\;$$

subject to EE>E (threshold), CC<T (maximum), $\sum_{i=1}^{3}\alpha_{i}=1$ and $\alpha_{i}\in \left[0,1\right]$.

Here, nodes die if their energy is less then E (threshold), and T (maximum) is the maximum range of node transmission.

#### 3.3.4. Clustering Steps

After the initialization of parameters, which is presented in the simulation setup (Section 4), the clustering phase consists of the following steps.

- Setting the Location and Velocity of the Particles
  The location and velocity of the particles are placed randomly in the following intervals, respectively: $\left[x_{min},\;x_{max}\right]$ and $\left[v_{min},\;v_{max}\right]$.The particle swarm size and harmony memory size (HMS) are initialized under the title of particle harmony memory (PHM). The initial PHM matrix [29], as shown in Equation (19), includes the number of random solutions generated to reach the optimal or near-optimal solution. Each row in the HMS×K matrix is a random solution, and the fitness function for each vector is calculated by Equation (18).
  (19) $$\left[\begin{array}{cccc} I_{1}^{1} & I_{2}^{1} & \cdots & I_{k}^{1}\\ I_{1}^{2} & I_{2}^{2} & \cdots & I_{k}^{2}\\ \vdots & \vdots & \ddots & \vdots \\ I_{1}^{HMS} & I_{2}^{HMS} & \cdots & I_{k}^{HMS} \end{array}\right]\left[\begin{array}{c} f_{\mathrm{clustering}\_1}\\ f_{\mathrm{clustering}\_2}\\ \vdots \\ f_{\mathrm{clustering}\_n} \end{array}\right]=\left[\begin{array}{c} F^{1}\\ F^{2}\\ \vdots \\ F^{n} \end{array}\right]$$
- Improving Harmony Vectors for PHM and Updating the Hybrid Matrix
  After PHM is defined, new harmony vectors $\left[I{{}^{\prime }}_{1},\;I{{}^{\prime }}_{2},\ldots ,\;I{{}^{\prime }}_{k}\right]$ should be produced to improve the harmony vectors of the PHM. Each component of the new harmony vectors is generated by harmony memory considering the rate (HMCR), and eventually an optimal candidate vector of PHM is modified by the pitch adjusting rate (PAR). In this step, the state-of-the-art method [29] uses the simple structure of HSA, but attention must be paid to variations in different stages of the search process. Additionally, the study in [34] demonstrated that HSA suffers from a slow convergence speed to achieve a global optimum; however, in [31], the dynamic version of HSA exhibited fast convergence at the start of the algorithm, converging to the global optimum in the near-final stages of the search process. Therefore, in this paper, each harmony vector (HV) of the PHM is obtained dynamically. The procedure of improving the harmony vectors for PHM is described in Algorithm 1 [31].
**Algorithm 1:** Pseudo code of Improving Harmony Vectors for PHM
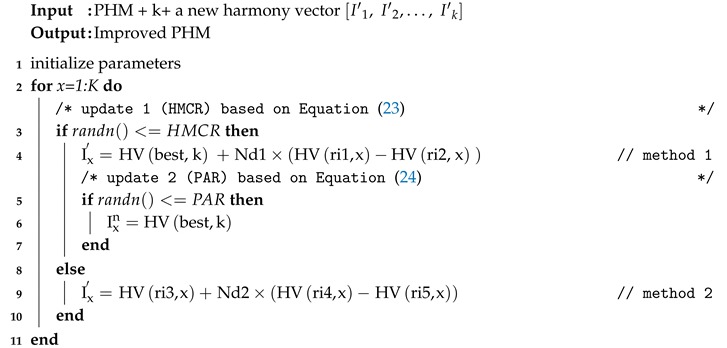

where Nd1 and Nd2 are normal distribution functions with (μ=0,σ=1), ri1 and ri2 are random integers between [1, HMS], ri3, ri4 and ri5 are random mutually exclusive integers between [1, HMS], the function randn() is a uniform random number in [0, 1], and parameter k is generated uniformly by a discreet random number in [1, K] [31]. After method 1 in Algorithm 1, if randn()≤ PAR, the harmony vector (HV) should be tuned or pitched (a term in music) according to the PAR. $I_{x}^{n}$ is defined as the closest node with more energy than current CH is the cluster. It is necessary to clarify that parameter best in update 2 as the index of the best HV inspired by the GHS method [45]. Next, updating the PHM is the last step in the HS algorithm [29]. To do this, if the fitness function of the new harmony vector ($I_{x}^{n}$) is better than the value of the fitness function of the worst harmony vector in PHM ($f\left(I_{x}^{n}\right)>f\left(HV_{x}^{worst}\right)$), the new harmony is included in the matrix and the current worst harmony of the PHM is excluded. Thus, the PHM is updated with this condition, and this step is stopped If the iteration of HSA is bigger than the maximum iteration.
- Updating the Location and Velocity of the Particles
  In this step, to reach the optimal location of the CHs, the PSO algorithm updates the location and velocity of the particles [2]. The best particle location (p_b) is determined by the minimum cost of the fitness function in a swarm particle [29]. The g_b, as the global best location, is determined by selecting the best solution of p_bs. In this paper, the nodes that harmonize with g_bs are taken, and the closest nodes to these harmonies form CHs. The velocity (V) and location (X) of the particles can be updated as follows [23,29]:
  (20) $$V_{\mathrm{xy}}\left(r+1\right)=\;\;\;\;\;\;\;\omega V_{\mathrm{xy}}\left(r\right)+r_{a}c_{a}\left(\;\;\;\;\;\;\;p\_b_{\mathrm{xy}}-X_{\mathrm{xy}}\left(r\right)\right)+r_{b}c_{b}\left(\;\;\;\;\;\;\;g\_b_{\mathrm{xy}}-X_{\mathrm{xy}}\left(r\right)\right)$$
  (21) $$X_{\mathrm{xy}}\left(r+1\right)=X_{\mathrm{xy}}\left(r\right)+V_{\mathrm{xy}}\left(r+1\right)$$
  where the index xy denotes the *x*th particles and dimension, $c_{a}$ and $c_{b}$ are acceleration constants, $r_{a}$ and $r_{b}$ are the random uniform amount in [0, 1], and factor ω is the inertia weight factor. Selecting a suitable value of ω creates a balance between exploration and exploitation [46]. In [29], the linear reduction of factor ω has a positive effect on the efficiency of the application of WSNs, while in [46], the authors proposed a nonlinear time-varying inertia weight formula that led to a faster convergence than the linear inertia factor used in [29,45]. Therefore, to enhance efficacy, we use the following high-order (∝) nonlinear formula:
  (22) $$\omega =\;\;\;\;\;\;\;\omega_{\max}-\left(\;\;\;\;\;\;\;\omega_{\max}-\;\;\;\;\;\;\;\omega_{\min}\right){\left(\frac{\mathrm{iter}}{r_{\max}}\right)}^{\propto }$$
  where iter is the current of iteration and $r_{\max}$ is the total number of iterations. Equation (22) [46] decreases nonlinearly from $\omega_{\max}=0.9$ to $\omega_{\min}=0.4$ and finds a suitable value at each round. The obtained results of [46] show that excellent performance is achieved when $\propto \;=1\;\;\;\;\;\;\;/\;\;\;\;\;\;\;\pi^{2}$ and the two constants $c_{a}$ and $c_{b}$—as cognitive and social parameters—have the value 2. The location and velocity of the particles are calculated and updated at each round of the proposed method and for each row in PHM. As mentioned above, the closest nodes to g_b will be the optimal CHs.
- Going Back to the First Step or Stopping the Algorithm
  When all the rounds of the clustering phase come to an end, the algorithm stops and a total number of *k* optimal CHs are determined for the routing phase. Otherwise, the algorithm goes back to the second step and repeats until it finds optimal or near-optimal solutions. However, before the next round starts, the values of HMCR and PAR in Algorithm 1 are required to be updated as follows [31]:
  (23) $$Update1:\mathrm{HMCR}\left(\mathrm{Iter}+1\right)=\;\;\;\;\;\;\;\mathrm{HMCR}.\min\;\;\;\;\;\;\;+\left(\frac{\mathrm{Iter}\;\;\;\;\;\;\;}{r_{\max}}\right)\times \left(\mathrm{HMCR}.\max\;\;\;\;\;\;\;-\;\;\;\;\;\;\;\mathrm{HMCR}.\min\right)$$
  (24) $$Update2:\mathrm{PAR}\left(\mathrm{Iter}+1\right)=\;\;\;\;\;\;\;\mathrm{PAR}.\max\;\;\;\;\;\;\;-\;\;\;\;\;\;\;\;\;\;\;\;\;\left(\frac{\mathrm{Iter}}{r_{\max}}\right)\times \left(\mathrm{PAR}.\max\;\;\;\;\;\;\;-\;\;\;\;\;\;\;\mathrm{PAR}.\min\right)$$
  These dynamic updates in the search process enhance the exploration capacity of the first stages and lead to the improvement of the local search in the last stages [31] because the variation of the HMCR value affects the diversity and convergence of PHM, with similar results for the PAR value in terms of the local and global optimum [34,41,42]. Hence, the initial values of Equations (23) and (24) are adjusted according to [31].

### 3.4. Routing Phase

In the routing phase, when the clustering phase is done optimally and a total number of k CHs are determined, the algorithm enters the routing phase to transmit the collected data. The routing phase in the proposed method is multi-hop routing, so it is necessary to specify the RNs in the sink path. The CMs send the information to their CH based on the Time Division Medium Access (TDMA) schedule, and the CHs pass the route to the sink based on the RNs [27]. To achieve this, only PSO is implemented, and the fitness function starts to construct the routing tree [39]. The transfer route leads to the formation of a routing tree [27,39]. If the tree is optimized more, this will have an important effect on the proper and quick transfer of the information.

#### Enhanced Tree Encoding

For the fitness function in this section (minF_routing), we use Equation (7), mentioned above [18], to determine the relay nodes (RNs). Next, the algorithms update the particle locations and velocities in the PSO. The RNs are responsible for transferring the gathered data to the sink. Considering the sensitivity of the points involved in a disaster area compared to other points, the information of these points needs to be transferred quickly; in order to build the routing tree, the PSO is performed again via the Base Station (BS) [39].

**Figure 2:**
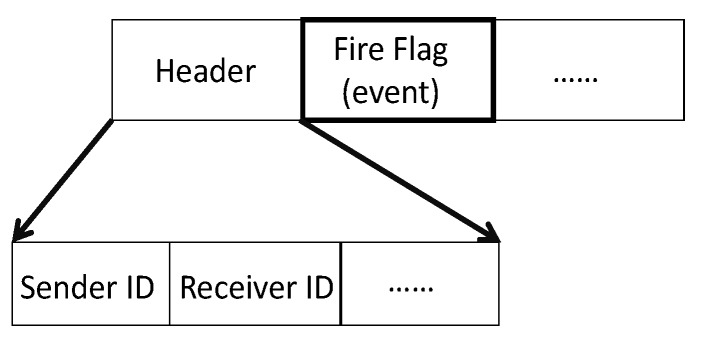
The new data packet format.

It should be noted that, by updating the location and velocity, the PSO increases the number of invalid paths [27]. Consequently, due to application, the number of invalid paths must be reduced, and valid paths must be detected quickly. To achieve this goal, the priority encoding/decoding approach of PSO has been successfully tested [27,39]. However, it is necessary to develop the tree encoding appropriate to the disaster areas. Thus, the new packet format with respect to the fire flag is presented in Figure 2.

**Figure 3:**
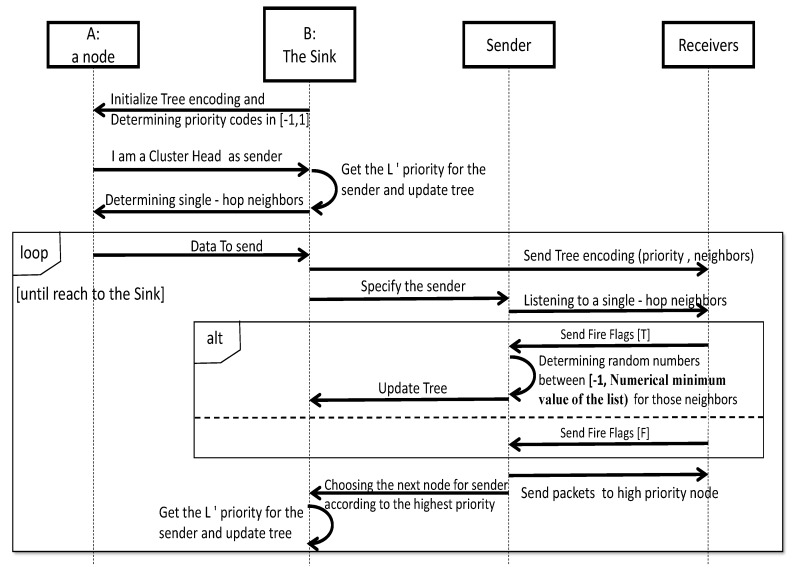
UML sequence diagram of tree encoding for each CH.

In Figure 3, a fire flag has been set up because the nodes are somehow aware of each other’s status and make a more accurate decision regarding whether to send data to each other on the basis of events. In disaster areas, the fire flag can take two Boolean values of T as a non-uniform event (the disaster state) and F as a uniform event (the normal state). In order to validate paths from the CHs to the sink, nodes (particles) are mapped onto a random-priority number between [−1, 1]. The path construction procedure is shown for each CH in the following the Unified Modeling Language (UML) sequence diagram:

For clarity, the process of the construction routing tree with a simple example is shown below.

**Figure 4:**
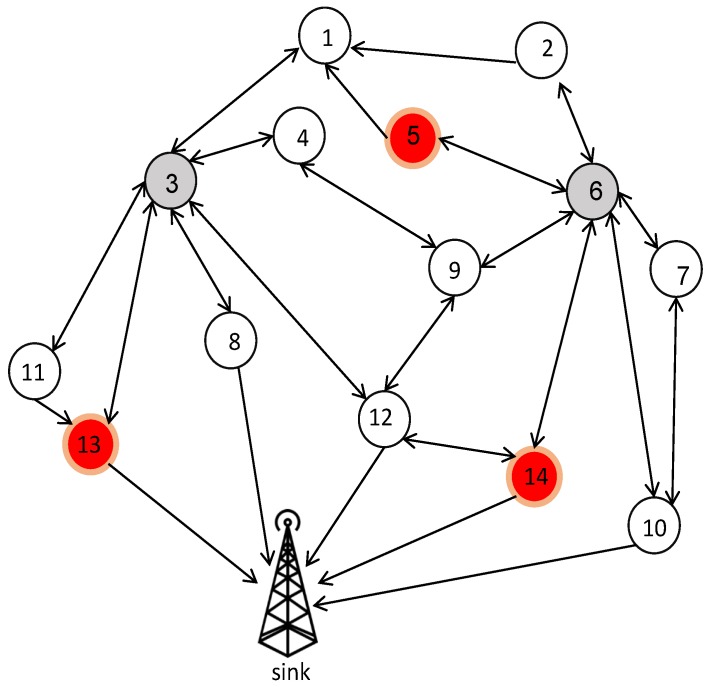
A random deployment of 15 nodes with two cluster heads of CH 3 and CH 6.

As shown in Figure 4, we have 15 nodes (with the sink and two CHs, 3 and 6) which are mapped onto the particles between [0, 14]. The sink has a tag of 0 with the highest priority H > 1, and nodes 5, 13 and 14 are in the fire range. The goal of all paths will be to reach the sink. For each CH, a list of 15 items is created based on the ID of the particles and their random priority. Next, each node, as the receiver identified by the sender, listens to its single-hop neighbors and chooses the next hop on the basis of the highest random priority generated between them.

**Figure 5:**
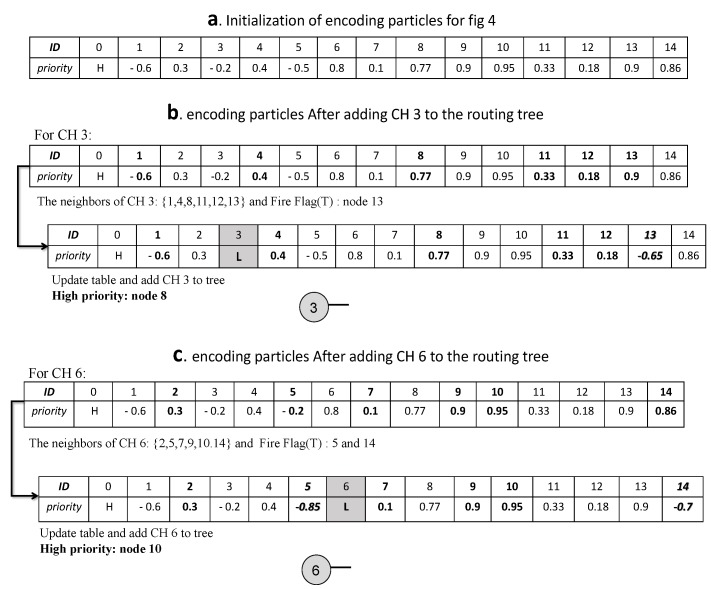
A simple example of modified tree encoding: (**a**) initialization of encoding particles for Figure 4 based on a random-priority number between [−1, 1], (**b**) encoding particles after adding CH 3 to the routing tree, and (**c**) encoding particles after adding CH 6 to the routing tree.

In the following, if the sender node takes a flag value of F when listening to its single-hop neighbors, then the next-hop is chosen based on the highest priorities of the single-hop neighbors. Otherwise, if the sender node takes the flag value of T, then the sender generates random priorities between $\left[-1,\;Numerical\;minimum\;value\;of\;the\;list\right]$ for the single-hop neighbors (updating encoding particles). In this way, the packets try to pass through more reliable paths. Next, for the next hop chosen, the penalty L (L < −1) is set so that it does not participate in the selection process again. The path formation based on modified tree encoding for each CH (3 and 6) is illustrated in Figure 5.

**Figure 6:**
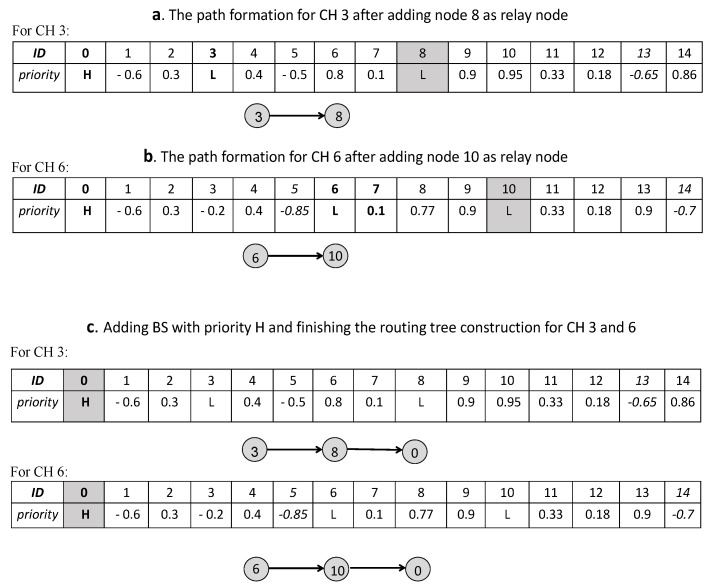
According to Figure 5, we continue the process of modified tree encoding: (**a**) the path formation for CH 3 after adding node 8 as a relay node (RN); (**b**) the path formation for CH 6 after adding node 10 as an RN; (**c**) adding BS with priority H and finishing the routing tree construction for CHs 3 and 6.

As observed in Figure 5, the value of the fire flag is returned to T for CH 6 from nodes 5 and 14, and also for CH 3 from node 13. Thus, random priorities between $\left[-1,\;Numerical\;minimum\;value\;of\;the\;list\right]$ are generated. Finally, choosing a single-hop neighbor based on the highest priority will construct the routing tree as shown in Figure 6.

According to Figure 5 and Figure 6, paths 3→8→0 and 6→10→0 are generated, and then the routing tree and optimal CHs are broadcast to all nodes by the sink. After this phase, the sleep scheduling of nodes is considered. Additionally, if CHs, CMs, and RNs are unable to send any data, the sink will go to the sleep state and save energy.

### 3.5. Overall Proposed Algorithm

The main pseudo-codes of the proposed algorithm are formulated in Algorithm 2.
**Algorithm 2:** Overall proposed pseudo-code (ECHSR)
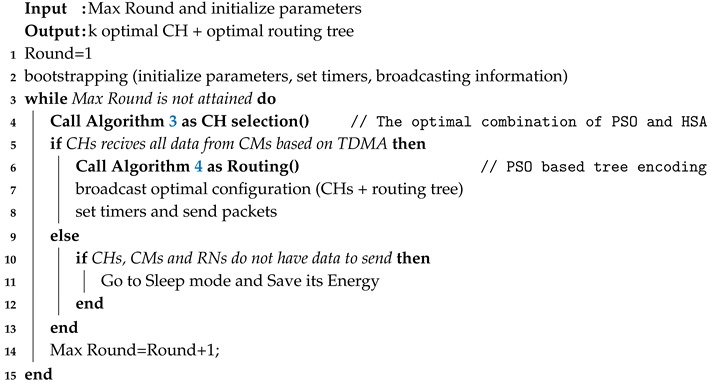


**Algorithm 3:** The pseudo-code of CH-selection()
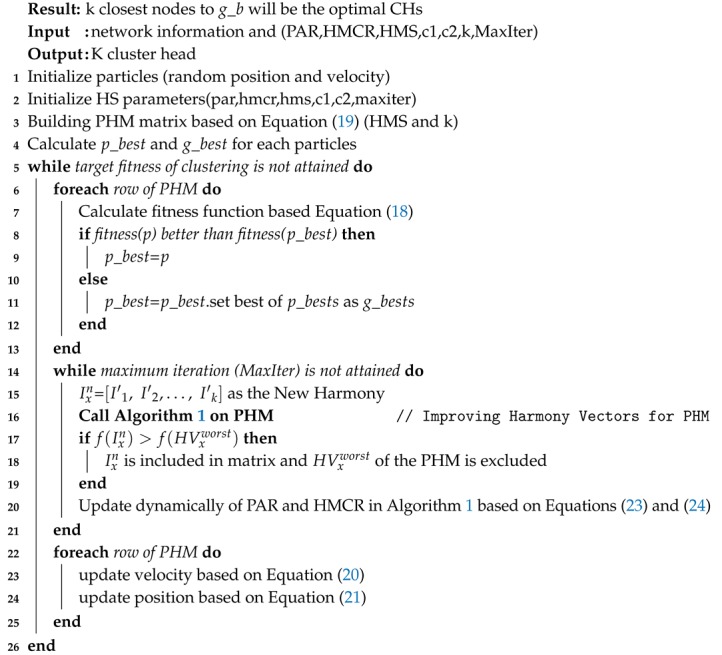


**Algorithm 4:** The pseudo-code of Routing()
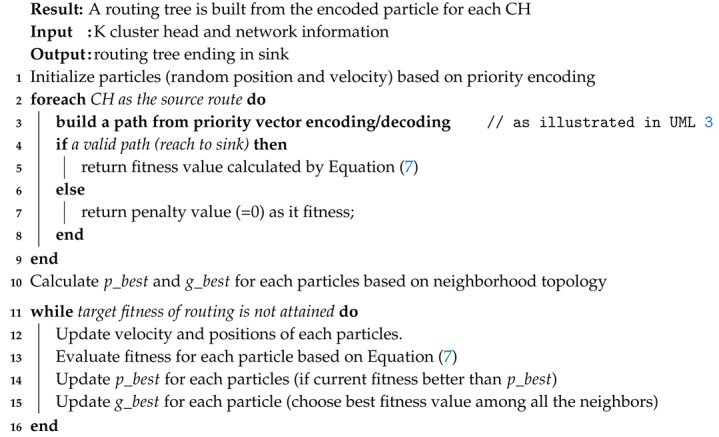


## 4. Simulation Results and Evaluations

**Figure 7:**
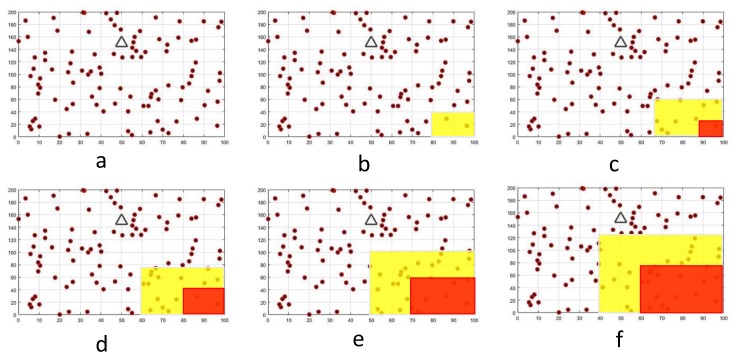
Simulation scenarios for forest fire in this work: (**a**) The normal state of the environment; (**b**) Fire smoke changes the state of the environment; (**c**) The fire starts in the forest; (**d**–**f**)— illustrates the spread of fire until 40 round of simulation.

In this section, our proposed method (the so-called ECHSR) is compared with the state-of-the-art works on PSO–HSA [29] and TPSO-CR [27]. Furthermore, we implement the classical LEACH algorithm [22] as an example to show why we must transition to evolutionary algorithms. However, we performed the simulation in terms of the overall residual energy, the number of live nodes, network coverage, and the packet delivery ratio (PDR) with four different sink locations (the coordinates (50, 150), (100, 100), (0, 100) and (50, 200)) and also repeated each simulation 10 times with forest fire scenarios (Figure 7). Moreover, to evaluate efficiency, we examined our protocol with a uniform distribution of events using the metrics of the average residual energy and PDR. To begin the comparison, the simulation setup is explained, and then the simulation results are presented and analyzed.

### 4.1. Simulation Setup

MATLAB 2017a was used for the simulation. The system on which MATLAB was installed and the simulation processes were conducted had the following properties: CPU: Inter Core i7, memory: 8 GB RAM and operating system: Windows 10 Enterprise-64bit. The parameters and their values are presented in Table 2. For the AWS method, we assign the same weights to the parameters in the simulation [27,38], $\alpha_{1}=\alpha_{2}=\alpha_{3}=0.33.$

To bring the results closer to the real-world situation and make the theories more practical, it is necessary that the simulation environment for scientific research does not deviate greatly from reality. Many works, such as [13,20], have been conducted on forest fire modeling and have investigated how they burn. Given the simulation’s capabilities, the computational complexity, and the difficulty of predicting the direction of fire, we decided to consider the speed and direction of the wind as being constant. By examining the previous works, we concluded that the fire burning model could be as shown in Figure 7; for instance, the NetLogo group implements forest fire models and confirms that fire spread can be random [47].

In this work, a forest fire spreads from one direction and expands to the center [47]. To show the validity of the results, in addition to the scenario shown in Figure 7, we performed another simulation (without fire) in Section 4.3. In our random model, the yellow area indicates the non-uniformity of the events and red areas show the burned forest and annihilated nodes. As shown in Figure 7, the forest fire scenario includes different processes: (a) from round 1 to round 6, the simulation shows the normal state of the environment; (b) from round 6 to round 12, there is a non-uniform event such as fire smoke, which is indicated in yellow; (c) from round 12 to round 18, forest fires occur, which are shown in red. Similarly, for the rest of the scenario—(d), (e) and (f)—Figure 7 shows the growth of the forest fire proceeding to rounds 24, 30 and 40 of the simulation, leading to more dead nodes.

### 4.2. Simulation Results

In this section, the criteria for the comparison and analysis of the results are the overall residual energy, active nodes, network coverage and PDR ratio.

#### 4.2.1. Overall Residual Energy

**Figure 8:**
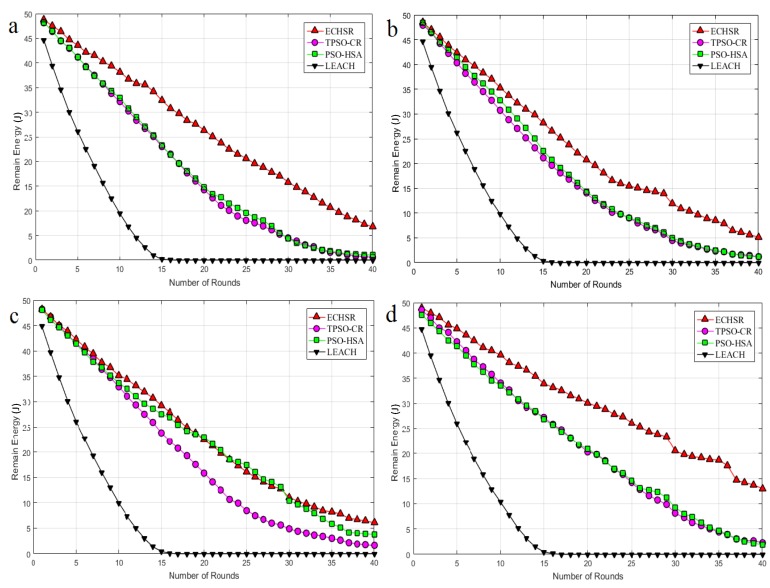
The residual energy for sink locations (**a**) (100, 100), (**b**) (0, 100), (**c**) (50, 150), and (**d**) (50, 200).

**Figure 9:**
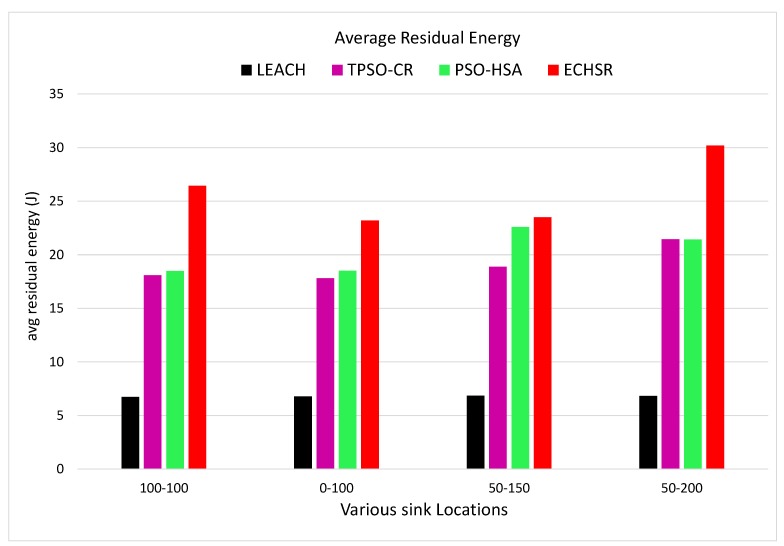
The average residual energy along with various sink locations.

Considering the limitation of energy nodes, one of the goals of this paper was to present an energy-efficient algorithm. The decrease in the overall residual energy for all nodes leads to an increase in the network lifetime. This shows how the energy consumption rate in all nodes is distributed uniformly and optimally in different rounds. The residual energy is calculated as follows:

(25) $$\sum_{n=1}^{N}E_{n,X}=E_{overall}$$

where $E_{n,X}$ is the overall energy consumption by a sensor node (n) after X rounds of data collection from the area-of-interest of the network, and $E_{overall}$ is determined as overall energy consumption by N number of total nodes after X number of simulation rounds of data collection in the network [42]. The mean of $E_{total}$ until round X is defined as the average residual energy in this paper. According to Figure 8 and Figure 9, although PSO–HSA [29], which is highly energy-efficient, is one of the most novel algorithms, this method could not compete with our method in forest fire scenarios (Figure 7).

As shown in Figure 8, because of the random selection of CHs, the residual energy of LEACH drops to zero in the early rounds (around 15 rounds). Until 40 rounds of simulation have passed, it can also be seen in Figure 9 that PSO–HSA has a better average residual energy than TPSO-CR because the efficient searching of HS complements the dynamic capacity of PSO and also improves the balance between exploration and exploitation. In other words, the proposed method has better energy dissipation distributions than other methods. Additionally, the proposed method is improved in terms of the overall residual energy by about 1.04–1.42 times compared to PSO–HSA, by about 1.24–1.46 times compared to TPSO-CR and by about 1.70–1.77 times compared to LEACH for the different sink locations according to Figure 8. The reasons for this improvement are the optimum improvement of the structure of PSO–HSA and the attention paid to non-uniform events in the routing phase.

#### 4.2.2. Active Nodes

**Figure 10:**
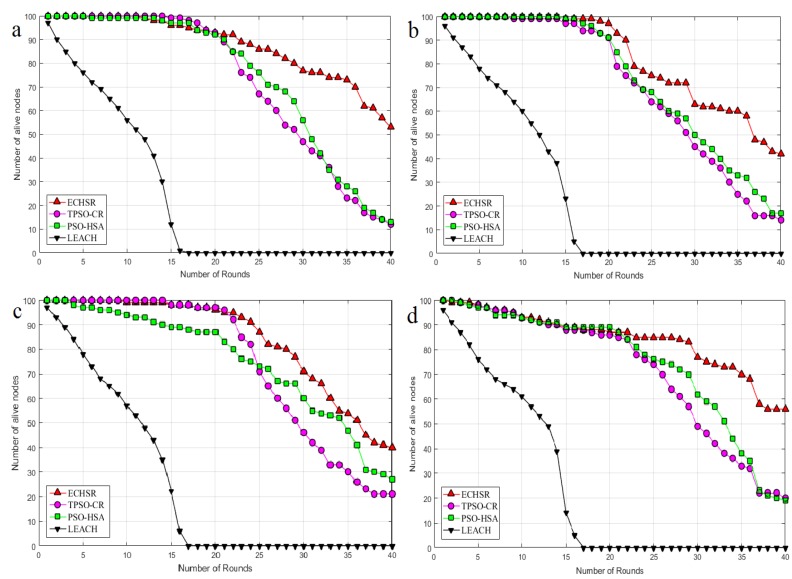
The number of live nodes for sink locations (**a**) (100, 100), (**b**) (0, 100), (**c**) (50, 150), and (**d**) (50, 200).

Another significant factor that directly affects the network lifetime is active nodes. Due to non-uniform events, the node energy involved in the disaster environments reaches zero after a while. The greater number of live nodes—or the reduction of the number of dead nodes—demonstrates that a better division of labor has taken place in terms of energy, and the CHs have optimally transferred the gathered data to the sink. In other words, the number of nodes increases the network lifetime that must be achieved to ensure network coverage. As shown in Figure 10, the proposed method in terms of active nodes outperforms PSO–HSA by about 16% to 36% and TPSO-CR by about 19% to 39% for the different sink locations. It can also be seen in Figure 10 that the LEACH algorithm also suffers as a result of the randomly chosen CHs in this application.

#### 4.2.3. Network Coverage

**Figure 11:**
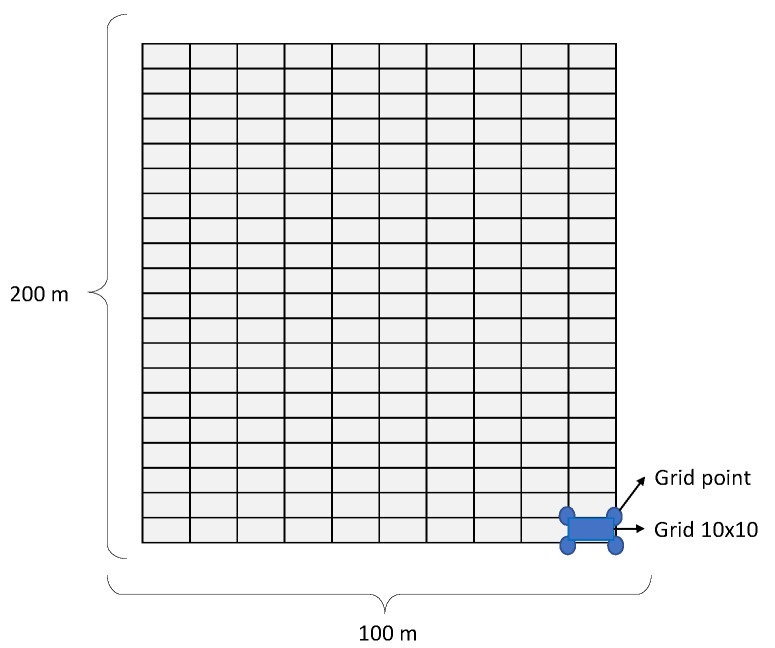
Dividing the area into smaller rectangles for coverage.

One of the most important factors of the quality of service (QoS) of WSNs is network coverage. The selection of the best set of CHs requires maximum coverage. The maximum area coverage detected by WSN in forest fire application is necessary, because these areas might not be appropriate for the nodes due to their uniform random distribution. In other words, as mentioned before, the goal of this measure is to reduce the number of non-CH nodes. In order to evaluate the network coverage properly, an environment of 100×200 is graded in squares of 10×10 (dividing the area into smaller rectangles), as shown in Figure 11. Thus, the maximum number of graded points is 231, and the number of grids is 200. The goal of this division is to minimize the number of uncovered grids.

**Figure 12:**
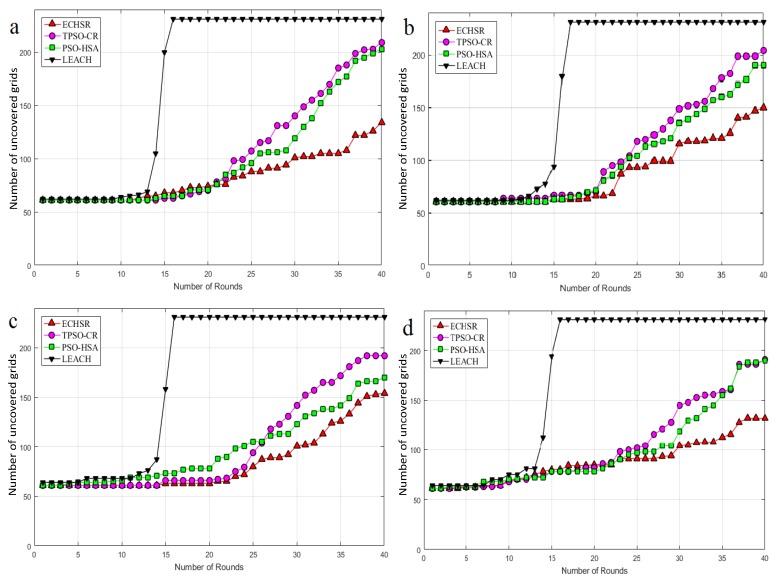
Network coverage for sink locations (**a**) (100, 100), (**b**) (0, 100), (**c**) (50, 150), and (**d**) (50, 200).

As can be seen in Figure 12, inspired by Figure 11, the proposed method outperforms PSO–HSA by about 1.08–1.32 times and TPSO-CR by about 1.18–1.34 times for the different sink locations. It is observed that the LEACH algorithm in round 16 has a network coverage of zero, which is much lower than the other methods. In general, the numerical results in Figure 12 demonstrate that the proposed method can cover the number of non-CHs more efficiently than PSO–HSA and TPSO-CR.

#### 4.2.4. Packet Delivery Ratio

In the network domain, the ratio of data packets that the destination (sink) receives to the total packets generated by sources (nodes) is called the packet delivery ratio (PDR). The improvement of this factor leads to an increase in the number of active nodes. Furthermore, the greater the number of simulation rounds, the more PDR will be reduced. The PDR for each round of simulation is calculated with Equation (26):

(26) $$\mathrm{PDR}=\;\;\;\;\;\;\;\frac{\mathrm{Number}\;\;\;\;\;\;\;\mathrm{of}\;\;\;\;\;\;\;\mathrm{packets}\;\;\;\;\;\;\;\mathrm{received}\;\;\;\;\;\;\;\mathrm{by}\;\;\;\;\;\;\;\mathrm{the}\;\;\;\;\;\;\;\sin k}{\mathrm{Total}\;\;\;\;\;\;\;\mathrm{packets}\;\;\;\;\;\;\;\mathrm{generated}\;\;\;\;\;\;\;\mathrm{by}\;\;\;\;\;\;\;\mathrm{nodes}}$$

**Figure 13:**
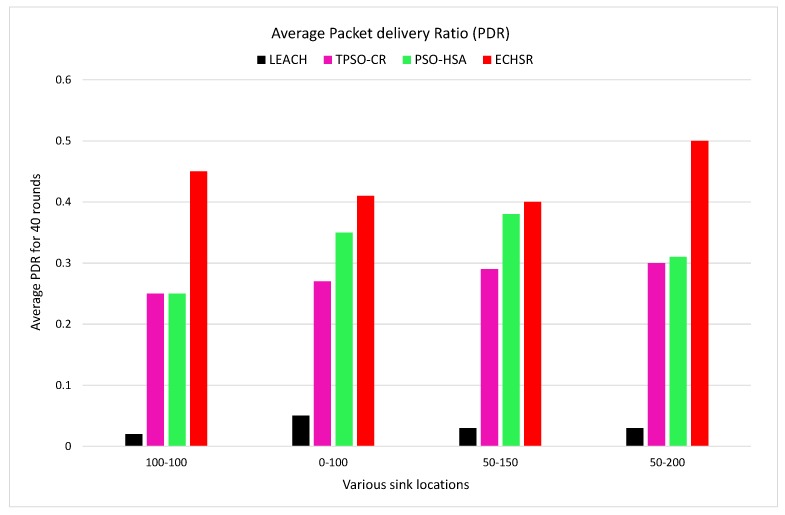
Packet delivery ratio (PDR) for various sink locations in forest fire scenarios and 40 rounds of the simulation.

As shown in Figure 13, considering that the worst link quality is removed by the fitness function (Equation (8)) and also that its routing tree is based on modified priority encoding, the proposed method has a higher average packet delivery ratio than other methods in 40 rounds of simulation. Furthermore, the numerical result demonstrates that PDR is increased in our method by about 1.07 to 1.38 times compared to PSO–HSA and by about 1.27 to 1.39 times compared to TPSO-CR for the different sink locations. It is clearly exhibited that LEACH has the lowest average PDR.

### 4.3. Uniform Distribution of Events

**Figure 14:**
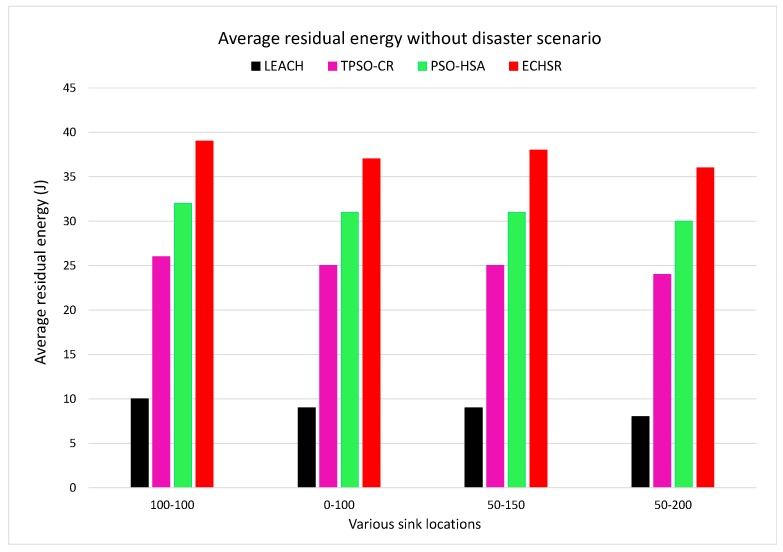
The average residual energy without forest fire scenarios for various sink locations.

We also tested the proposed method without a forest fire scenario (stable condition) in terms of the metrics of the average residual energy and packet delivery ratio (PDR) for various sink locations. In other words, across the entire network, events are uniformly distributed in each round of the simulations. According to the obtained results for the uniform distribution of events, the last node dies in the proposed method at about 1850 to 1900 rounds, which is superior to the state-of-the-art method [29], where this occurs at about 150 to 200 rounds. Up to 1900 rounds of the simulation, as shown in Figure 14, our protocol experiences the maximum average residual energy and consequently enhances the network lifetime compared to other methods.

**Figure 15:**
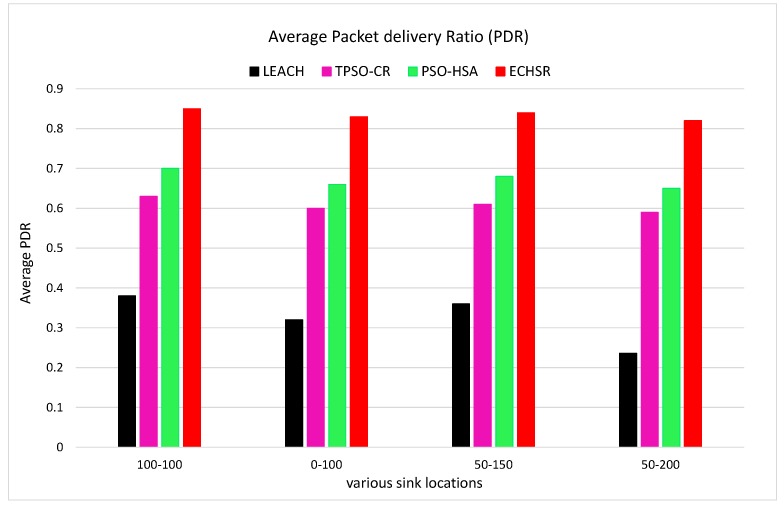
PDR without forest fire scenarios for various sink locations.

Unlike the forest fire scenarios (Figure 7), with the changing position of the sink, there is no obvious difference in the efficiency of the algorithms. However, in order to evaluate the quality of clustering links and improvements in the packet transmission, we performed the average packet delivery ratio (PDR) analysis in Figure 15 based on various sink positions. Figure 15 clearly shows that the proposed method has the highest average PDR compared to other methods.

## 5. Conclusions and Future Work

In this work, energy-efficient cluster head selection and routing methods based on evolutionary algorithms were proposed for safety-critical WSN applications, focusing on forest fire. We have targeted energy efficiency as a major issue in such IoT networks. To overcome this challenge, an improved hybrid PSO–HSA-based CH selection technique with a novel fitness function including energy-efficiency, cluster closeness, and network coverage criteria were proposed. We employed PSO to select RNs with a fitness function including energy-efficiency and communication link quality, which resulted in the development of a modified tree encoding method based on a new data packet format. We validated the efficiency of the proposed method for a forest fire scenario compared with the related works, considering different sink node placements. By considering different performance metrics, such as the overall residual energy, the number of live nodes, network coverage, and the packet delivery ratio in simulation evaluations, we could make the following conclusions: (1) the proposed algorithm in a disaster area is much better than the state-of-the-art methods [27,29]; (2) the placement of the sink node affects the performance of the algorithm; (3) since the variation of HMCR and PAR parameters affect the fast convergence of the algorithms, it appears that these parameters may change dynamically; (4) probabilistic algorithms such as LEACH [22] cannot perform properly in a disaster application; and (5) in scenarios where events occur uniformly, our algorithm improves the network lifetime compared with other methods.

In future work, we are planning to design efficient MAC protocols based on this application to prevent long delays and extend simulation scenarios, especially for heterogeneous networks.

## Figures and Tables

**Table 1:** Comparing different clustering protocols in Wireless Sensor Networks (WSNs) with our proposed method. LEACH: low-energy adaptive clustering hierarchy; TEEN: threshold-sensitive energy efficient sensor network protocol; PSO: Particle Swarm Optimization; HSA: Harmony Search Algorithm; CH: cluster head. TPSO-CR: Two-tier PSO protocol for Clustering and Routing. iCSHS: Integrated Clustering for Cuckoo Search and Harmony Search.

Reference	Clustering Schema	Converge	Energy-Efficient	Cluster Stability	Event Awareness	Routing	Clustering Objective
LEACH [22]	Random Distributed	No	Low	Low	No	single-hop	Changing CH based Prob./Random
TEEN [40]	Probability Centralized	No	Medium	Low	No	single-hop	Changing CH based Threshold
[23]	PSO-based Centralized	Low balanced	Moderate	High	No	single-hop	Energy Efficiency + Min Distance + Node Hop count
[41]	HSA-based Centralized	Low balanced	Medium	Low	No	single-hop	Energy Efficiency + Min Distance
TPSO-CR [27]	PSO-based Centralized	Moderate balanced	Moderate	High	No	multi-hop	Energy Efficiency + link Quality + Coverage
PSO-HSA [29]	PSO and HSA Centralized	Moderate balanced	High	High	No	single-hop	Energy Efficiency + Min Distance
iCSHS [42]	Cuckoo-based Distributed	Moderate balanced	Moderate	Low	No	multi-hop	Energy Efficiency + Node degree + Intra Distance + Coverage
This Paper	PSO and HSA Centralized	High balanced	very High	High	Yes	multi-hop	Energy Efficiency + Cluster Closeness + Coverage

**Table 2:** Parameter settings for simulation.

N	AN	MR
N	Number of nodes	100
m×m	Sensor field region	100 m × 200 m
E0	Initial energy of a nodes	0.5 (j)
K	Number of CHs	5
DP	Data packet length	4096 (bits)
DA	Energy data aggregation	5 (nJ)
[Xmin, Xmax]	Particle position	[0, 200]
[Vmin, Vmax]	Particle velocity	[0, 200] (m/s)
[c1min, c1max] and [c2min, c2max]	Acceleration constants	2
Eamp	Multipath fading transmitter amplifier energy	130 (pJ/bit/m2)
Eelec	Transmission and receiving energy	50 (nJ/bit)
Efs	Free space transmitter amplifier energy	10 (pJ/bit/m2)
Max Round	Maximum number of rounds	40 rounds
Sw	Swarm size	15
Iter	Number of iterations	5
HMCRmin and HMCRmax	Harmonic memory considering rate	0.8 and 0.9
PARmin and PARmax	Pitch adjustment rate	0.1 and 0.9
$\omega_{\max}$	Maximum values of inertia weight	0.9
$\omega_{\min}$	Minimum values of inertia weight	0.4
Base Station (BS)	Various sink locations	(0, 100), (100, 100), (50, 150), (50, 200)
